# A Mixed-Method Assessment of Drivers and Barriers for Substituting Dairy with Plant-Based Alternatives by Danish Adults

**DOI:** 10.3390/foods14152755

**Published:** 2025-08-07

**Authors:** Beatriz Philippi Rosane, Lise Tjørring, Annika Ley, Derek Victor Byrne, Barbara Vad Andersen, Susanne Gjedsted Bügel, Sophie Wennerscheid

**Affiliations:** 1 Department of Nutrition, Exercise and Sports, University of Copenhagen, Rolighedsvej 26, 1980 Frederiksberg, Denmark; shb@nexs.ku.dk; 2 Sino-Danish College (SDC), University of Chinese Academy of Sciences, 380 Huaibeizhuang, Huairou District, Beijing 101408, China; derekv.byrne@food.au.dk (D.V.B.); barbarav.andersen@food.au.dk (B.V.A.); 3 Queen Mary’s Centre, University of Copenhagen, Øster Farimagsgade 3, Building 30, 1353 Copenhagen, Denmark; alt@samf.ku.dk; 4 Department of Food-Nutrition-Facilities, FH Münster, Corrensstraße 25, 48149 Münster, Germany; 5 Food Quality Perception and Society Team, iSense Lab, Department of Food Science, Faculty of Technical Sciences, Aarhus University, Agrofood Park 48, 8200 Aarhus, Denmark; 6 Department of Nordic Studies and Linguistics, University of Copenhagen, Emil Holm’s Channel 2, 2300 Copenhagen, Denmark; sophie.wennerscheid@hum.ku.dk

**Keywords:** dairy attachment, plant-based alternatives to dairy, plant-based foods

## Abstract

The market for plant-based alternatives to animal foods has increased rapidly in the past decade, mainly due to consumer demand. Little evidence is available regarding nutritional impacts, drivers, and barriers to using these products as substitutes for animal foods in real-life conditions. This pilot study followed 16 Danish adults (30 ± 11 years old; 11 females) for 4 weeks with substituting milk, cheese, and yogurt with plant-based analogues to dairy (PBADs) and assessed their drivers and barriers to applying the intervention with a mixed-method approach. PBADs are constantly compared to their animal counterparts, both regarding product characteristics, such as price and sensory properties, as well as cultural roles and subjective memories. The mixed methods showed dairy attachment, price, and taste were the main barriers to consuming PBAD, while changes in life and social circles were drivers (qualitative data). As for the liking of PBADs, plant-based yoghurt was the preferred intervention product (73.5/100, *p* < 0.05), followed by plant-based drinks (65.9/100), while plant-based cheese was the lowest rated (47.9/100, *p* < 0.05). As for dietary changes, a lower average intake of sugars, saturated fatty acids, cholesterol, calcium, phosphorus, and zinc was observed after the intervention. Additionally, this study describes the attachment of the study population to milk and dairy products. It shows that choosing dairy is beyond nourishment but is connected to tradition, culture, pleasure, memories, and a sense of belonging. In contrast, there is no history or attachment to PBADs.

## 1. Introduction

The offer of plant-based alternatives to animal protein has grown rapidly in the past decade, driven by an increased interest from consumers to reduce their intake of animal products, motivated by animal welfare or environmental concerns [1]. Alternatives to dairy products, in particular, have become popular also because of allergies or intolerances to cow’s milk [2,3]. These products, notably plant-based drinks or alternatives to milk, are often perceived as healthier compared to cow’s milk by PBD consumers [4]. However, consumers tend to prefer animal dairy due to familiarity, taste, and price [2].

It is not clear, however, what the health impacts of the use of plant-based alternatives as substitutes for their animal counterparts are. Most studies in the area are limited to the nutritional composition of the products and fail to consider the effects of a substitution in the context of diets. There are studies using modelling to hypothesize these changes [5,6,7,8], but no clinical trials in real conditions.

This gap in knowledge is particularly interesting to study in Denmark, which has one of the world’s largest consumptions of milk per capita [9], and a strong traditional dairy industry [10]. It is commonly agreed that milk and dairy products are part of Danish culture [10]. However, little has been written about the cultural embedding of dairy products in Danish people’s lives.

It is generally recognized that food habits are influenced by a variety of social and cultural factors [11,12,13]. Whereas Buhl highlights the historical evolution of Danes’ relationship to dairy, other research points to the influence of everyday social relations on food choice [14,15,16], culturally developed food habits [12,17,18], and socio-economic factors [19,20,21].

Because cultural, socio-economic, and psychological factors influence food choices, an interdisciplinary approach is required to understand the complexity of consumers’ perceptions [4]. Using a mixed-methods approach, our primary aim is to investigate the drivers and barriers to substituting milk and dairy with plant-based analogues among participants in a pilot intervention study in Copenhagen, Denmark. As a secondary aim, this study investigates the changes in diet composition during the intervention. This explorative pilot study builds a framework for future intervention studies and raises insights for settings with a similar strong dairy background as Denmark.

## 2. Materials and Methods

### 2.1. Study Design

**Figure 1:**
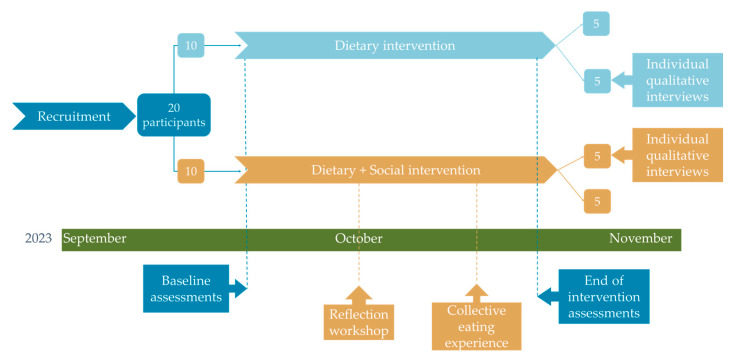
Study design flowchart. “Baseline assessments” and “End of Intervention Assessments” include a 3-day dietary record and questionnaires. After the recruitment phase, participants were asked to answer demographic questions when signing the consent forms.

This exploratory study consisted of a 4-week dietary intervention study using mixed methods to conduct the study and assess data. At recruitment, participants were assigned to one of two different groups: either dietary intervention or dietary + social intervention (see Figure 1). Both groups were subjected to a dietary intervention substituting milk and dairy for PBADs, later reported in Section 2.3. Five participants in each group were invited to an individual qualitative interview (described in Section 2.5.3). Moreover, participants in the ‘dietary + social intervention’ group engaged in two social activities throughout the study, as described in Section 2.5.1 and Section 2.5.2. The quantitative assessments, later listed in Section 2.4, were conducted at baseline (BL) before starting the dietary intervention and again at the End of the Intervention (EI).

The mixed-method approach was used to capture more information about individuals’ food choices. While quantitative methods are useful for objectively assessing habits, perceptions, and intentions, qualitative methods enable a more in-depth understanding and reflection on individuals’ opinions.

The Capital Region’s Regional Scientific Ethics Committee was notified of the study on 16 August 2023 (Journal no.: F-23048555), and, since no biological material was collected, it was not considered a clinical trial according to Section 2 of the Committee Act. The study was considered to be based on questionnaires and interviews and, therefore, does not require authorization from the Committee to be conducted (Section 1(4) of the Committee Act).

Although the Committee’s approval was not necessary, the study adhered to Good Clinical Practice standards of ethics. Despite the lack of biological samples and the low risk of the intervention, these standards are designed to protect participants’ health. One example is that the intervention was considered risky for vulnerable populations, influencing the inclusion and exclusion criteria. Moreover, all participants freely signed a declaration of consent to participate in the study. They were aware that they could withdraw their participation and ask to have their data deleted at any time until publication. Data was stored and processed according to the EU’s General Data Protection Regulation.

### 2.2. Recruitment and Study Population

Participants were recruited using announcements on the University of Copenhagen news boards and social media platforms (Facebook and Instagram). People interested in participating in the study were asked to fill in an interest form with their age, gender, and contact information. They were asked screening questions to ensure they met the inclusion criteria (self-report format). Inclusion criteria were (a) be a healthy adult, (b) be between 18 and 60 years old, (c) have been raised in a Danish context, and (d) be a fluent Danish speaker. They should also (e) be a frequent consumer of dairy products and (f) be willing to substitute milk, yogurt, and cheese for plant-based analogues completely. Exclusion criteria were (a) pregnancy or breastfeeding, (b) allergy to the intervention products’ ingredients (i.e., soy, oat, pea, or cashew nuts), (c) be following a special diet for disease management and/or restrictive vegetarian diet, or (d) use of any medication that affects their palate or appetite.

At the information meeting, in person or over the phone, potential participants were asked if they were available for the social activities and again about the inclusion criteria. Allocation to the different intervention groups was not random, prioritizing participants’ availability to attend the social activities (see Section 2.1). All participants also agreed to potentially be assigned to qualitative interview groups.

A total of 20 participants were recruited. They were all Danish, living in the Greater Copenhagen area. Participants were, on average, 29 ± 8.7 years old, and five were male. During the dietary intervention, two female participants dropped out for personal reasons not related to the study, and one female was excluded after becoming ill, which drastically affected her eating habits. One female dropped out because she believed the intervention was too demanding and required too much effort to consider her food choices. At the end of the study, 16 participants (30 ± 11 years old) successfully finished the interventions, and the group provided the basis for data analysis and the results of this article.

### 2.3. Dietary Intervention

Participants were asked to maintain their habitual diets except for milk, yogurt, and cheese, and to avoid other dairy products when possible. Weekly, participants were offered plant-based drinks (oat or pea), plant-based yogurt (soy-based), and plant-based alternatives to cheese (cashew nut-based). Each participant received as many plant-based analogues to dairy (PBADs) as needed to meet their habitual intake of the substituted dairy products. For example, if they typically drank 2 L of milk weekly, they would be given 2 L of plant-based drinks. As a marker of compliance, participants were asked to return the empty packages weekly when getting new products. They were also asked about their feelings regarding the intervention, any difficulties they experienced, and their preferences for the products. The products provided for the intervention diet are listed and described in Supplementary Materials SM 1.

### 2.4. Quantitative Assessment Methods

#### 2.4.1. Dietary Assessments

Participants’ habitual diets were assessed using a 3-day dietary record at baseline. Participants were instructed to fill out a form with the day and time of each meal and to specify in as much detail as possible the food eaten and the amounts. This registration was performed on three consecutive days, one of which was a weekend. The dietary assessment was repeated during the last week of the dietary intervention while participants were still eating the plant-based dairy alternatives.

#### 2.4.2. Questionnaire: Attitudes, Perceptions, and Motivation for Consumption

Participants’ attitudes towards eating more sustainable foods, motivations for consuming more sustainable foods, and perceptions and well-being of plant-based dairy alternatives were assessed using an online questionnaire. The questionnaire was developed to explicitly evaluate drivers and barriers to consuming sustainable foods and PBADs and consumers’ perception of PBADS [22]. They answered these questions using a visual analogue scale ranging from 0–100, where zero stands for “Don’t like it at all” and 100, “Like extremely”. Other variations of anchors were used depending on the question, such as “Not at all important/Extremely important”.

Additionally, three questions regarding social behavior were included in the questionnaire (see Supplementary Materials SM 2) and answered on a 5-point Likert scale. Participants were asked to answer the questionnaire at baseline and the end of the dietary intervention. Well-being was also measured one week after the start of the intervention. The results of the well-being questionnaire are not included in the present paper’s findings. The questionnaire was sent to and answered by participants in the Research Electronic Data Capture (REDCap) [23] hosted by the University of Copenhagen.

### 2.5. Qualitative Assessment Methods

Qualitative assessment methods were based on three events (see Figure 1), with two being group events with group interactions and individual reflections and one being individual semi-structured interviews. All events are described below.

#### 2.5.1. Reflection Workshop

The purpose of the reflection workshop was to encourage the participants to reflect individually and collectively on their eating habits and to collect data on their experiences, memories, thoughts, and habits regarding dairy consumption and the transition to plant-based alternatives. The reflection workshop consisted of four distinct parts, where the participants reflected on childhood memories related to dairy, their perceptions regarding dairy cows’ lives, the connection between social life and food choice, and sustainability. During the workshop, the participants produced individual texts and discussed the four themes with each other and in plenum. The inspirational material and descriptions of the exercises are available in Supplementary Materials SM 3. The individual texts and the notes taken during plenum discussions and interactions were used as data.

#### 2.5.2. Collective Eating Experience

The purpose of the collective eating experience was to give the participants an experimental sensory and social experience of a dinner to encourage the participants to discuss dairy products and plant-based products in new ways. During the collective eating experience, a three-course dinner was served. Each course consisted of three options to choose from: entirely plant-based, containing dairy, or containing meat. The participants were invited between the courses to discuss, reflect upon, and sense the food through parlor games, literary readings, and coordinated discussions. A video recording of the collective eating experience was used as data.

#### 2.5.3. Semi-Structured Qualitative Interviews

The qualitative interviews aimed at capturing individual opinions and experiences regarding dairy consumption and the transition to plant-based alternatives. The interviews were conducted in Danish and took place in the participants’ homes. The reason for conducting the interviews in the homes was to make the participants feel relaxed, inspire new questions spurred by the home setting, and give a deeper understanding of the participants.

The overall topics covered in the interviews were motivation for participating in the pilot project, the experience of replacing dairy products with plant-based alternatives, childhood experiences of food, opinion about the new products, and views on the relationship between food choice and the green transition. The interviews were semi-structured to capture what was important to the participants by following relevant themes they brought up, rather than adhering strictly to the interview guide. The interview guide for the qualitative interviews is available in Supplementary Materials SM 4. The interviews were transcribed and used as data.

### 2.6. Data Analysis

#### 2.6.1. Dietary Data

The nutritional composition of participants’ diets was assessed by adding the dietary records to a nutritional software platform (Vitakost^©^, Vitakost Aps 2024). In addition to nutritional composition, foods were classified into food groups and quantified in portions. This process is described in Supplementary Materials SM 5. The investigation of food groups and portions examines if the intervention would cause further dietary changes, as the PBADs have a different nutritional composition than that of dairy, namely, drinks are less calorie-dense, and all PBADs tend to be lower in protein [24,25].

#### 2.6.2. Questionnaire Data

Data from the questionnaires were analyzed in Microsoft Excel Version 2501 and R Studio (R version 4.4.3 (2025-02-28 ucrt)). Continuous variables were analyzed using the arithmetic average and standard deviation. As data was not normally distributed, the Mann–Whitney U test was used to compare differences between assessments (BL and EI) and variables. Categorical variables (e.g., multiple-choice questions) had frequent answers counted. Due to the small sample size and the exploratory nature of the study, no statistical tests were conducted on the categorical variables.

#### 2.6.3. Qualitative Data

Qualitative data from the reflection workshop, collective eating experience, and interviews were subjected to narrative analysis and coded in the qualitative analysis program NVivo, version 14. Topics for the analysis were selected and validated based on the frequency of appearance across the participants in the data material.

The most common topics raised fell within the categories of motivations, perceptions of products, the influence of social relations and experiences, social/physical settings, and life phases and landmark events.

## 3. Results

### 3.1. Demographics

The study population (*n* = 16) was predominantly female (*n* = 11, 68.75%) and young (mean age, 30 ± 11 years), unequally distributed amongst the four intervention groups, as seen in Table 1. Most participants live in small households, either alone or with another person, with an annual income of up to 225,000 Danish kroner (*n* = 9).

### 3.2. Quantitative Data

#### 3.2.1. Dietary Changes

At baseline (BL), participants reported high consumption of dairy products, especially milk (305 g/d) and yogurt (282 g/d), and very little intake of meat (about 5 g/d), as seen in Table 2. Also at BL, some participants already reported using plant-based alternatives to dairy and meat.

Towards the end of the intervention period (EI), participants adhered to the restriction of not consuming dairy products, including milk, yogurt, and cheese. The intake of total dairy products at BL was aligned with the intake amount of total PBADs at EI. Average consumption of the plant-based version of milk and yogurt at EI was close to the intake of dairy versions at BL. The lowest alignment was observed for the average intake of dairy cheese and plant-based cheese, where 50% of the amount of dairy cheese consumed at baseline was consumed as plant-based cheese at EI.

Participants reported an increased intake of fish and shellfish, fats and oils, nuts and seeds, and whole-grain cereals. There was also an increase in the proportion that vegetables and fruits contributed to total energy intake (from 4.55 to 5.62 E%; Table 2).

Table 3 shows the nutritional composition of participants’ diets at both assessment time points and the Danish National Intake’s average for reference. There was a pronounced reduction in the intake of sugars, saturated fatty acids, cholesterol, calcium, phosphorus, and zinc. Additionally, there was a higher intake of dietary fiber, selenium, and vitamin D (Table 3).

Macronutrient intake was within the national average from 2011–2013 [26]. However, for sugars, the study population ate more than twice the national daily average at BL (102.9 g/d vs. 49 g/d), and despite a decrease in intake at EI (from 102.9 g/d to 83.2 g/d), the average consumption remained higher than the national average after the intervention.

#### 3.2.2. Questionnaire Results

Participants were asked about their liking of PBADs, with separate questions for plant-based drinks (milk alternatives), plant-based yogurt, and plant-based cheese. Plant-based cheeses were the lowest scored, with an average liking of 47.9 ± 25.9 (where 100 = like extremely), significantly lower than plant-based drinks (65.9 ± 17.2; *p* < 0.05). Plant-based yogurt was the preferred product, with an average liking of 73.5 ± 22.4 (100 = like extremely), and significantly higher than the previous two product categories (*p* < 0.05).

When asked how important were different characteristics of plant-based and climate-friendly foods on their motivation to consume these foods (see Supplementary Materials SM 6), participants indicated similar (*p* > 0.05) high importance to price (66.2 ± 29.9 BL, 68.9 ± 25.2 EI, 100 = very important), healthiness (79.5 ± 17.1 BL, 78.2 ± 12.7 EI), information about the product (69.5 ± 21.8 BL, 63.7 ± 17.9), sensory properties (77.4 ± 21.6 BL, 75.5 ± 11.2 EI), use in everyday dishes, easy to use/prepare, and being able to incorporate in their habitual diets. Regarding the challenges observed in climate-friendly products, price was the most frequently indicated (62.5% at BL and 81.2% at EI), followed by sensory properties (56.2% at BL and 62.5% at EI). Of the least commonly picked were climate impact (6.2% at BL and 0% at EI), variety (12.5% at both BL and EI), and familiarity (18.7% at BL and 12.5% at EI). There were no significant differences between BL and EI assessments.

### 3.3. Qualitative Data

The results of the qualitative data from the reflection workshop, collective eating experience, and interviews are overlapping and have been grouped into the following topics based on the coding of the data material: motivations, perception of products, the influence of social experiences and relations, social/physical settings, and life phases and landmark events. The results on each of the topics are presented below, and the data source is specified for each topic.

#### 3.3.1. Motivations

In the interviews, the participants were asked to describe their motivation to be part of the pilot project. The answers varied between being curious and wanting to try something new, finding it an easy way to try new products, and enjoying taking part in research projects. Several participants also mentioned that they study food-related subjects at university, which was their main reason for participating in the pilot project. When asked about their motivation to change diets in general, several mentioned a wish to be healthier, and some mentioned animal welfare and a concern for the environment.

However, many participants also pointed out in the interviews that they felt confused and conflicted as to what the right thing to do is. For example, some participants described some of the plant-based products as ultra-processed products, which they did not perceive as healthy. Often, participants’ motivation stagnated due to such considerations.

#### 3.3.2. Perceptions of Products

The most common topic raised in plenum by the participants themselves during the reflection workshop and collective eating experience was their experience and perception of the plant-based alternatives. Taste and price of the products dominated the discussions, and typical topics were whether one liked/disliked the taste of particular products and whether one perceived the prices of the products as fair or unfair.

Apart from the taste and price, the participants were concerned with the texture (most often perceived as worse than the dairy products), the involuntary sugar intake (some plant-based drinks and yogurts contained sugar), the plant-based products being perceived as too synthetic, and the lack of potential for substituting cheese and cream in a good way (taste and texture-wise).

In the reflection workshop, participants were asked to reflect on the origin of the products through an exercise of writing a brief text from the perspective of a dairy cow. Notably, most of the texts produced described industrial dairy settings but emphasized that the cow’s life was meaningful because of the value humans place on its milk. Only a few texts acknowledged underlying anxieties, for example, a cow’s worry about being removed if her milk yield decreased.

#### 3.3.3. The Influence of Social Experiences and Relations

Data from the interviews and the written texts on childhood memories about dairy from the reflection workshop showed that all participants had clear memories and experiences of dairy products being an integrated part of their diet and something that naturally belonged in the fridge. Most significant was the experience of school milk and the different associations of the types of milk offered. The participants had various memories of dairy products, ranging from buying ice cream from the ice cream car that drove around on the weekends to eating buns with thickly spread butter at birthdays and eating special cheeses and food with cream as a special treat, as exemplified in this citation:

“When we had guests, we always had special cheeses and not just the ordinary cheese from the supermarket.”

ID35, female, 23 years old

Although the majority of the experiences and memories were positive, it was not always the case, as the following citation shows:

“I didn’t want to drink school milk. I remember my friends said it caused pimples, and that influenced me a lot. My schoolteacher tried to make me drink it because my parents paid for it.”

ID26, female, 23 years old

The majority of the participants expressed concern about not wanting to impose a burden on family and friends by requiring special food made for them. It was a practical burden in the sense that it caused family and friends to change the way they normally cooked or to cook something in addition to what they would normally cook, but it was also a social burden. Asking family and friends to make special food was described as socially uncomfortable and potentially creating conflicts. It was also described as requiring strength and a surplus of energy to insist on eating something other than what the others ate. For this reason, many of the participants opted for a flexible choice of food, where their food habits in their homes were different than their food habits in other surroundings, as exemplified in the following quote:

“I went to a restaurant with my family-in-law, and we were ordering a variety of dishes to share. I just didn’t have the energy to argue for the vegan options and having to explain myself, so I just had what the others had.”

ID41, female, 31 years old

Many participants expressed a wish to avoid social conflicts. However, as described above, this was not always a barrier to eating plant-based foods; it could also be a driver. For example, one participant explained how he had stopped eating beef because his new girlfriend did not like it, and it was easier to cook something they both enjoyed. Another participant described how his daughter asking for plant-based food had made him transition to a more plant-based diet.

#### 3.3.4. Social and Physical Settings

Many participants described how the physical setting of the kitchen and the social life connected to the physical setting influenced their food habits. For example, one participant explained how living in a student dormitory with a shared kitchen had made her less prone to trying new foods:

“We were so many sharing the kitchen and there was never space enough to do proper cooking. I always ended up making something quick and hurried out of the kitchen afterwards. It was a period of my life when I didn’t give food much thought. Afterwards, I moved into my own apartment, and now I find myself enjoying cooking, and I often experiment with new types of food and recipes.”

ID29, female, 24 years old

On the other hand, another participant had the opposite experience:

“I moved to a student dorm with a shared kitchen. We took turns cooking for each other. There were always some vegetarians present, and it was just easier to make a vegetarian meal for everyone than making two meals. I got a lot of vegetarian dishes into my food repertoire that way.”

ID22, male, 24 years old

Generally, the participants expressed a positive association between having a nice kitchen (whether that meant physically (for example, spacious and clean) or socially (for example, sharing it with friends/family)) and their wish to spend more time and mental energy on cooking.

#### 3.3.5. Life Phases and Landmark Events

Many of the participants mentioned how entering student life had been a driver for changing food habits. Vegetarianism and veganism were experienced as common and automatically accommodated for when, for example, cooking for/with friends. Moving to the city and becoming a student also meant new friendships and relations, which inspired and exposed participants to other types of food habits. One participant described how moving to the United States and entering new social relationships had opened her eyes to new food possibilities and made it easier for her to become a vegetarian.

Not just new life phases, but also particular events were described as having an effect on food habits. For example, one older participant described how he had experienced falling ill on a tourist trip to Paris and subsequently found out that it was due to high blood pressure. The incident shocked him and led him to change his food habits significantly in order to lower his blood pressure through dietary changes:

“After the episode in Paris, I decided to eat less meat and fatty food and more vegetables. It makes sense to me to avoid medicine when I can lower my blood pressure through dietary changes.”

ID45, male, 57 years old

### 3.4. Honeycomb Overview of Drivers and Barriers for Consuming PBADs

**Figure 2:**
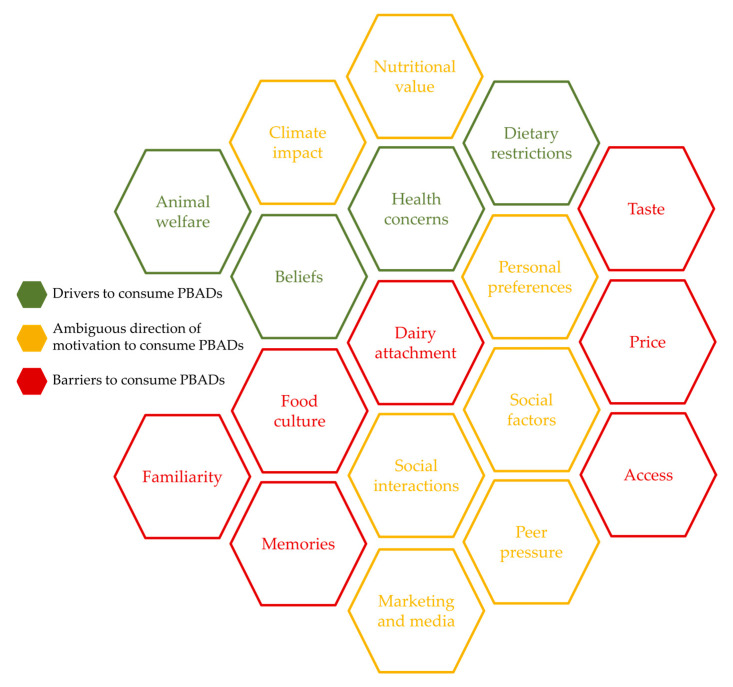
Honeycomb overview of drivers and barrier aspects for consuming plant-based alternatives to dairy in the study population. Green (e.g., animal welfare) represents drivers to consume PBADs. Red (e.g., dairy attachment) represents barriers to PBADs consumption. Yellow (e.g., climate impact) represents aspects where the directions are ambiguous.

In Figure 2, we display an overview of the drivers and barriers aspects for consuming PBADs in the study population. Given the mixed-method approach, the results from quantitative and qualitative methods were combined to assess these aspects. This overview is performed in the form of a honeycomb to illustrate the complexity and interplay of different factors. Drivers are colored in green and barriers in brown, while ambiguous aspects, that is, were seen as both barriers and drivers, were colored in blue. Dairy attachment is placed in the center, showing it as a strong factor, constantly present when choosing to consume PBADs.

The honeycomb illustrates that the drivers and barriers aspects of consumers’ lives are not opposite. In fact, they are often ambiguous, having different effects depending on the individual and the situation of food choice.

## 4. Discussion

Based on the mixed-method approach, the results will be discussed, using the themes commonly observed and comparing both quantitative and qualitative data. First, we propose the concept of dairy attachment to describe the complex relationship between the study participants and dairy products. Second, the discussion touches upon participants’ perceptions of PBADs. In this topic, the sensory attributes of PBADs were mentioned as a barrier to consumption, together with conflicting thoughts about the health and environmental impacts of these novel products. Third, it discusses the dietary changes caused directly and indirectly by the intervention. Fourth, it explicitly mentions the impact of social interactions and life phases on food choice and choosing to consume PBADs. Fifth, we list the positive perceptions and memories towards cow’s milk and dairy as a barrier to consuming PBADs. Lastly, we discuss limitations, such as the small sample size, and strengths of the mixed-methods approach as a comprehensive analysis of drivers and barriers to the consumption of PBADs and the pioneering intervention with PBADs.

### 4.1. Dairy Attachment

In the present study, the role of dairy in dietary habits and the use of cow’s dairy as a reference to discuss plant-based alternatives were constant. Our results describe that drivers and barriers to increasing plant-based dairy analogues are not limited to product characteristics (e.g., sensory, price, and nutrition) but are also related to hedonic perceptions (pleasure), culture, and memories, both positive and negative. The study population also had a high habitual intake of milk and yogurt, at 305 g/d and 282 g/d, respectively, higher than the average intake of Danish adults of 317 g/d of milk and milk products [26]. These results show a high degree of dairy attachment.

Other researchers have not conceptualized dairy attachment, but we propose that it is similar to meat attachment [27]. Dairy attachment comprehends the complexity of the role of dairy in the diets of this study population. This attachment also relates to how hard it would be for participants to abstain from dairy consumption for longer periods of time. Our concept of ’dairy attachment’ draws inspiration from attachment theory in the sense that it is an emotional bond [28] and a secure base [29]. We see dairy attachment as developing through repeated interactions and experiences within a particular cultural group [30]. The concept is also inspired by studies of food identity [31] in the sense that attachment is seen as closely linked to the formation of identity. Despite not being mentioned in the literature before, dairy attachment is likely high in Danish and other Nordic populations due to the historically high intake of dairy [9] and the role of the dairy industry in the region’s economy [10,32].

Dairy attachment was present across all drivers and barriers, particularly because plant-based products are often marketed as alternatives to dairy and are therefore naturally always compared to dairy. It seems that even if plant-based alternatives were equally good or better than dairy in terms of taste, price, environmental impact, and nutrition, they still cannot replace the cultural role of dairy in a Danish context.

The cultural attachment to dairy has a long history. Danish food historian Bettina Buhl traces milk consumption in Denmark back to the Bronze Age and highlights key developments, such as technological innovations and the cooperative movement of the 1880s, the introduction of school milk programs after World War II, and the distribution of household pamphlets in the 1960s that guided the incorporation of quality dairy into everyday meals [33]. These developments have made it “deeply ingrained in us that milk is healthy and something we should have every day.” [33]. The texts produced in the reflection workshop on memories of dairy products show how the participants had experienced dairy products as naturally being part of their everyday food. Although in various ways and to various degrees, it seemed like all participants from an early age had been socialized through family life into dairy consumption. Mette A. E. Kim-Larsen [34,35] has described milk and dairy consumption as a marker of being Danish and how families with non-ethnically Danish adopted children struggle with their lactose intolerance and therefore the impossibility of consuming dairy.

However, the attachment to dairy may be changing. Buhl emphasizes that milk consumption today is declining due to environmental concerns and changes in drinking habits, and a report by the Agriculture and Food Council [36] shows that dairy consumption per capita has declined. Also, it is questionable whether the TV milk advertisements from the 1990s featuring a Danish top model bathing in milk would be as socially acceptable today as then. Plant-based products have the potential to take over the dairy’s role but still lack cultural integration, history, and a place in childhood memories.

### 4.2. Perceptions of Plant-Based and Animal Dairy

Sensory properties (e.g., taste and texture) and price were the most often cited barriers to the consumption of PBADs, both in group interactions and in the questionnaire answers. Participants felt that most PBADs used were not as good as dairy products, except for yogurt, and mentioned that PBADs are more expensive than their animal counterparts. In contrast, most of the participants shared positive memories of dairy products, and only a few participants shared unpleasant childhood memories, such as being forced to drink the school milk they did not want to drink. Similarly, previous studies have found taste and price to be major drivers for consuming not only dairy but also other plant-based alternatives to animal foods [37,38,39].

Although price is a fixed number, it can be perceived in a variety of ways. What is perceived as expensive for some may be experienced as fair or cheap for others, depending on, for example, one’s dedication to climate-friendly food, socio-economic status, and whether the product is perceived as a luxury or standard food. A study by Kalyva and colleagues (2024) showed that young people in Greece are more willing to value perceived environmental impact, the climate crisis, and the social signaling potential when purchasing food.

The majority of the participants in the present study were relatively young (30 ± 11 years old) and could be expected to be willing to pay more for climate-friendly products like plant-based cheese, based on the results from Kalyva and colleagues [40]. This was not the case in the present study. As the taste of the cashew cheeses provided in the intervention was perceived as relatively good compared to the other plant-based cheeses, it could be that their price was considered ‘extremely high’ and not just a bit higher. It could also be a general perception of the population, as plant-based alternatives are more expensive than their animal counterparts [41,42]. In the study of Martínez-Padilla and colleagues with consumers and non-consumers of plant-based drinks, all respondents perceived the alternatives to milk as expensive [43].

Additionally, there was the complexity of perceiving a product as climate-friendly, as an imported and processed cashew cheese was not necessarily perceived as climate-friendly. Concerns for the environment and health were mentioned in the interviews as motivators to change diet in general, aligned with the motivation aspects to eat climate-friendly food (Supplementary Materials SM 6). Concurrently, previous consumer studies mentioned the same factors as motivators for the consumption of plant-based alternatives to dairy products [3,4].

Participants seemed to perceive that the intervention with PBADs made their diets more climate-friendly, but also mentioned an internal conflict regarding these products, not knowing if consuming PBADs was the right choice. Some mentioned that the PBADs are highly processed foods and, for that reason, did not perceive them as healthy. A recent study of Danish consumers [43] showed that perceiving plant-based alternatives to milk as highly processed was inversely correlated to the likelihood of being a consumer of these products. This discussion was not part of the focus of the present study but indicates that the processing level of these novel foods is taken into account by consumers when choosing whether to consume these products and should be incorporated into future consumer research.

Concern for animal welfare was also mentioned in the interviews as a motivator to change diet. Interestingly, most participants in the reflection workshop pictured cows in industrialized settings as happy cows in the reflection text about dairy cows’ lives. Considering that just before this writing exercise, the participants had been urged to critically reflect on the dairy industry, allowing for negative perspectives to be brought forth, this makes it even more significant that the participants chose to write the stories about a cow’s life in a positive way.

A clear connection emerges when comparing the cow perspectives in the reflection text to participants’ other writings on childhood memories of dairy and reflections on the sustainability of dairy. Those (the majority) with positive dairy-related memories tended to imagine cows in humane and sustainable conditions. In contrast, participants with less positive memories of dairy products often viewed the cow’s experience more critically and were more open to shifting toward plant-based alternatives. It is important to notice that animal welfare is a broad concept. Although most participants associated industrialized cows with happy cows, there might be other elements of the production chain that they were critical of (for example, slaughtering).

### 4.3. Dietary Changes

Aligned with the barriers and challenges reported by the participants, dietary data showed that participants did not do a one-to-one substitution of dairy products with PBADs. Specifically, the EI data showed a lower average intake of PBADs than of dairy products at BL. That could be a consequence of participants generally not liking the sensory properties of the PBADs as much as they like dairy products. In particular, taste and texture were identified as issues by both the questionnaire and the qualitative data. This was more apparent with the plant-based cheeses, with an average liking of 47/100 (significantly lower than other PBADs) and an intake 50% lower than that of dairy cheese at baseline (Table 2).

Participants’ EI diets were higher in dietary fiber and selenium, both likely due to an increased intake of whole-grain cereals [44]. Notably, there was an increase in Vitamin D3 intake due to more frequent fish consumption during the dietary assessment period (Table 3 and Supplementary Materials SM 7).

At EI, participants had lower sugars, saturated fatty acids, cholesterol, calcium, phosphorus, and zinc intake. The nutrients with reduced intake are a consequence of the intervention, as plant-based dairy alternatives have lower saturated fatty acids, no cholesterol or vitamin B12, and lower calcium, phosphorus, and zinc [45,46], even though some products were fortified with calcium and vitamin B12. Despite the fortification, the average vitamin B12 intake was higher at the end of the intervention assessment due to some participants’ intake of organ meats (e.g., liver pate) and energy drinks (Supplementary Materials SM 7).

The results of the intervention contrast with prior studies on diet modelling [5,6,7,8]. Unlike the models, the intervention has not observed a decrease in protein, iodine, and riboflavin intake, as expected. Contrastingly, the observed lower intake of calcium and vitamin B12 was expected. These divergencies in results could be due to differences in the study populations and baseline diets, but also because the intervention was not a controlled simulation as diet models, but free-living individuals that could, as so happened, not follow the intervention completely with a one-to-one substitution of dairy products with PBADs. Moreover, the participants could make further changes to their diets that were not predicted.

This study also aimed to investigate if participants would naturally change other food groups’ intake to compensate for the changes in nutrient intake, such as protein and energy, caused by the intervention. It was not possible, however, to pinpoint whether a specific food group intake increased (i.e., fish and shellfish) due to the intervention or by coincidence due to the small sample size and short time of intervention (4 weeks).

### 4.4. The Influence of Social Relations on Eating PBADs

Social relations were a driver and a barrier to transitioning to more plant-based food. Preserving relationships with family and friends was a high priority for the participants, and often, they chose to make compromises in social settings and abstain from their own food preferences. They were aware that eating differently affected their relationships and that they potentially risked being criticized and talked negatively about, creating distance from others and, at worst, undermining the relationship.

Consuming food is not a neutral act but invariably social and laden with meaning [16]. Food can define a boundary between persons and become a marker of social differentiation [47,48]. However, food does not just have the potential to weaken social relationships but also to strengthen them. By eating the same food, you create togetherness. In the example of the parent choosing to cook plant-based food for the daughter to meet her wish lies an attempt to strengthen their relationship. For the participants, their food choices were not just a matter of what they liked to eat but also a matter of navigating socially.

The social interactions between participants were a driver and a barrier in the study itself, and not just in the participants’ own lives. During the reflection workshop and the dinner experience, we observed the conversations that took place and how participants related to each other socially. It was clear that both reinforcements and impairments took place. For example, there were participants who confirmed each other in the correctness of eating less meat and drinking less milk, but there were also participants (particularly one) who were critical about transitioning away from dairy, arguing for the importance of the nutrient values of dairy. The critical perspectives of one participant were not necessarily adopted by the other participants, but made an impression on them, which was seen in the way that they all chose to bring up this one participant in the individual interviews.

Although it was not possible to track a dietary change based on participating in the social track compared to the non-social track, data from the qualitative interviews showed that participating in the social track had strengthened the participants’ reflections about the transition from dairy to plant-based products.

Data from the interviews showed several instances of the social/physical setting of the kitchen influencing food choices. In the results section, two quotes highlight the physical and social boundaries that a shared kitchen brought to two participants. The examples interestingly show how the same physical setting enables different types of social life, which is then again connected to food habits. This is in line with Fox [49], who points out that the physical setting is closely connected to the social when it comes to food consumption.

Where we cook and where we eat has been redesigned over time to fit social needs, whether that be the social aspiration of having a dining room like the upper class [49] or the open kitchens accommodating for changed gender roles or changed parent/child relationships in the family [50]. It also goes the other way around. The physical setting influences social life and the perception of the food being eaten [51]. In the examples with similar kitchens but different social lives, one social/physical setting became a hindrance while the other enabled the development of new food habits. Although the result may be indeterminate, the examples show that drivers and barriers are not clear-cut. The examples also remind us that food choices are not just individual matters but part of complex social and physical integrations that the individual is not necessarily aware of, nor has the autonomy to affect.

Life changes were additionally mentioned as drivers of dietary changes, such as moving to another city and the beginning of student life. Similar results have been reported before in the review by Zhang and Chang [52]. The authors highlight how changes in personal life affect consumers’ choices. The same study also points out that young people are more interested in trying new things, as new interests can be developed through travel, moving, and exposure to new cultures.

Common to all the participants experiencing a new life phase was the destabilizing effect such a new life phase had on them, whether it was due to travels, becoming a student, or being triggered by falling ill. Destabilizing one’s life created moments of openness to new ways of life, which allowed new habits to take form. Turner [53] describes this state of destabilization as a liminal period characterized by disorientation, but also the possibility of new perspectives, scrutiny of one’s values, and the building up of a new identity. During such a period, when one’s life is temporarily undone, one is particularly prone to change. In the last example with the health scare episode of falling ill in Paris, the participant reported a motivation to change his diet, aligned with previous studies [54,55].

### 4.5. Strengths and Limitations

The study had both strengths and limitations. To the authors’ knowledge, this is the first study to substitute milk and dairy products with plant-based analogues. Despite the small sample size, this study contributes to discussing whether PBADs are suitable substitutes for dairy foods by testing the substitution under real-life conditions and using PBAD products available for purchase. Additionally, the mixed-method approach allowed a better and often complementary assessment of drivers and barriers to consuming PBADs.

As a limitation, we acknowledge the high dropout rate (20%, 4 out of 20) and lack of randomization, which did not allow proper testing of the effects of the social activities as additional interventions. Moreover, the dietary intervention was quite short (only four weeks) and may not have had enough time to impact nutritional status, which was not assessed. We also acknowledge the limitations of the age group, with most participants being young people. Other age groups may experience life conditions that could have influenced the outcome of the study. Additionally, the sample was mainly female, which decreases the power of generalization as females tend to be more health-conscious and open to vegetarian and vegan diets.

The temporary aspect of the intervention was a strength as well as a limitation of the study. It was a motivation for the participants since it was not life-binding but appealed to their curiosity about trying something new (that they were already interested in). Furthermore, the fact that it was a temporary intervention made it easier for people to engage in social life, as participants felt greater understanding and acceptance from their peers when they could explain their changed food habits as ‘being part of an experiment’ rather than being ‘for real’. The latter tended to be assumed as a major change in identity, which was perceived by participants as more difficult for peers to accept and get used to, potentially creating discomfort in social relationships.

For the development of future intervention studies, it may be beneficial to engage more with the social aspects of temporality and the motivational factor inherent in experimental designs. However, the temporary aspect also posed limitations in the sense that reality was lacking. Because the study was time-constrained, the participants felt it was a short-term ‘experiment’ rather than ‘real’ life changes, lacking a feeling of obligation to change beyond the intervention period. There may be potential in developing the link between experimental design and permanent change in future studies.

## 5. Conclusions

Drivers and barriers to consuming PBADs in this study population depended on other aspects and the situation in which the choice was to be made. As shown in the honeycomb overview, several aspects of food choice are ambiguous and can be both drivers and barriers to the same individual.

As an explorative study, the present work builds a foundation for future intervention studies substituting dairy and other animal foods with plant-based substitutes. The complexity of the theme evidences the need for cross-disciplinary work and mixed-methods assessments of consumer perceptions towards plant-based foods and motivations to change diets.

Regarding the characteristics of plant-based products, the main barriers to consumption were taste and price, while health concerns were the main drivers. Additionally, the high processing level confused participants about whether the products were healthy.

In this study, we propose the term dairy attachment to describe Danish adults’ complex relationship with milk and dairy products. In Denmark, dairy intake is linked not only to nutrition and taste but also to tradition, culture, pleasure, memories, and a sense of belonging to the community. In this context, it is not enough for plant-based alternatives to dairy to be as good or better than dairy, as consumers do not have a history, memories, or attachment to PBADs, while dairy attachment is high.

## Figures and Tables

**Table 1:** The study population overview is distributed by intervention groups and social demographic characteristics.

Intervention Group	N of Participants	Gender	Age (Years; Mean ± SD)
Diet intervention + social activities + qualitative interviews	4	2 females 2 males	27 ± 3.7
Diet intervention + social activities	5	3 females 2 males	28 ± 8.5
Diet intervention + qualitative interviews	4	3 females 1 male	39 ± 16.3
Diet intervention	3	3 females	27 ± 3.1

**Table 2:** Average daily intake of different food groups in grams and in portions, with intake adjusted per 10 MJ/d in the diet.

	Grams/d		Portions/d	
**Food Group**	**Baseline**	**End of Intervention**	**Baseline**	**End of Intervention**
Total daily energy intake	-	-	-	-
Fruits and Fruit Products	144.65	145.50	1.99	2.38
Vegetables	216.76	195.73	5.12	4.52
Herbs and Spices	4.38	1.81	1.12	0.74
Legumes	51.14	26.79	0.74	0.34
Potatoes and Potato Products	59.18	23.69	0.61	0.24
Nuts and Seeds	10.40	24.64	0.65	1.53
Whole-grain cereals	111.96	145.61	2.65	2.83
Cereals and Pseudocereals	172.18	158.53	3.01	1.97
*Dairy (total)*	489.29	25.79	7.26	0.67
Milk	305.56	0.00	2.43	0.00
Yogurt	282.29	9.52	0.41	0.05
Cheese	65.19	8.33	3.39	0.40
Other Dairy Products	50.12	7.93	1.03	0.22
*Plant-based Dairy Alternatives (total)*	34.94	485.95	0.22	3.95
Plant-based Milk Alternatives	26.06	271.99	0.14	1.55
Plant-based Yogurt Alternatives	0.00	181.69	0.00	0.91
Plant-based Cheese Alternatives	0.00	29.05	0.00	1.45
Other plant-based products	2.60	3.21	0.08	0.13
*Meats (total)*	58.91	55.71	1.74	1.22
Red Meat	5.21	5.24	0.04	0.05
White Meat	28.00	34.38	0.26	0.41
Processed Meat	25.70	16.10	1.44	0.77
Meat Substitutes	7.92	2.62	0.67	0.22
Fish and Shellfish	13.67	38.57	0.19	0.90
Eggs	15.52	15.95	0.32	0.32
Fats and Oils	4.12	6.81	0.41	0.68
Sweets	43.60	26.79	4.08	2.49
Salty Snacks	6.46	1.79	0.29	0.07
Sauces	30.33	26.82	0.93	1.07
Non-Alcoholic Beverages	1571.79	1447.69	7.99	7.33
Alcoholic Beverages	349.40	96.87	1.60	0.52

**Table 3:** The nutritional composition of participants’ diets at baseline and towards the end of the intervention is normalized for a daily intake of 10 MJ. E% represents the contribution of the macronutrient to total energy intake. The Danish National Nutritional Intake is on the right for reference.

	Baseline		End of Intervention		Danish National Intake (2011–2013) *	
Total Average (per 10 MJ)	Mean ± SD	E%	Mean ± SD	E%	Results	E%
Total energy intake (MJ)	10.4 ± 2.8		9.0 ± 2.5		9.8 ± 3.1	
Protein (g)	89.3 ± 16.7	14.6	83.2 ± 15.5	15.8	88.0 ± 28.8	16.0
Carbohydrate (g)	279.2 ± 41.7	45.7	282.3 ± 33.5	53.6	239.0 ± 82.0	42.0
Sugars (g)	102.9 ± 40.9	36.9	85.5 ± 36.3	30.3	49.0 ± 38.4	8.0
Dietary fibres (g)	30.5 ± 11.9		44.7 ± 14.2		22.0 ± 7.9	24.0
Fat (g)	86.7 ± 14.2	30.9	90.0 ± 13.7	37.2	96.0 ± 36.9	36.0
Saturated fatty acids (g)	35.2 ± 9.7	12.5	22.4 ± 6.2	9.2	39.0 ± 16.1	14.0
Cholesterol (mg)	188.1 ± 121.0		144.2 ± 124.3			
Omega 3 (g)	1.7 ± 1.8		2.0 ± 1.6			
Calcium, Ca (mg)	1138.0 ± 388.5		919.7 ± 237.7		1166.0 ± 353.0	
Iron, Fe (mg)	9.2 ± 3.5		11.2 ± 4.4		11.9 ± 2.1	
Iodine, I (μg)	91.4 ± 34.4		179.8 ± 393.6		263.0 ± 155.0	
Magnesium, Mg (mg)	337.2 ± 86.8		362.5 ± 146.3		400.0 ± 81.0	
Phosphorus, P (mg)	1303.4 ± 272.7		952.8 ± 277.3		1634.0 ± 291.0	
Salt, NaCl (g)	5.6 ± 1.8		6.0 ± 1.3			
Selenium (μg)	29.6 ± 9.6		40.4 ± 18.0		55.0 ± 14.6	
Zinc, Zn (mg)	8.5 ± 1.8		6.9 ± 2.1		12.7 ± 2.3	
Vitamin B2 (mg)	1.5 ± 0.6		1.3 ± 0.6		1.8 ± 0.5	
Vitamin B12 (μg)	2.9 ± 1.9		3.4 ± 3.1		7.0 ± 3.3	
Vitamin B12 (added) (μg)	0.0 ± 0.0		0.9 ± 1.2			
Vitamin C (mg)	99.9 ± 57.8		103.8 ± 62.9		123.0 ± 65.0	
Vitamin D3 (μg)	0.0 ± 0.8		1.8 ± 2.9			

* Danish National Intake Survey (2011–2013) [26].

## Data Availability

The original contributions presented in this study are included in the article/Supplementary Materials. Further inquiries can be directed to the corresponding authors. Quantitative data will be available upon reasonable request. Qualitative data will not be available to protect participants’ identities.

## Supplementary Materials

The following supporting information can be downloaded at: https://www.mdpi.com/article/10.3390/foods14152755/s1; SM 1: Plant-based products used in the intervention; SM 2: Social questions/Questionnaire additional questions; SM 3: Description of the reflection and writing workshop on dairy attachment and plant-based alternatives; SM 4: Semi-structured qualitative interview guide; SM 5: Food group classifications and standardized portion sizes; SM 6: Results of motivation to eat climate-friendly. SM 7: Top 3 food sources of nutrients; SM 8: Total energy intake and energy consumption per food group; SM 9: Participants self-reported overall liking for PBDA and frequency of consumption. References [56,57,58,59,60] are cited in the Supplementary Materials.

## Abbreviations

The following abbreviations are used in this manuscript:

PBAD	Plant-based alternative to dairy
BL	Baseline
EI	End of Intervention
SM	Supplementary materials
