# Supplementary material for: A Mixed-Method Assessment of Drivers and Barriers for Substituting Dairy with Plant-Based Alternatives by Danish Adults
Source: Foods. 2025 Aug 7;14(15):2755. doi: 10.3390/foods14152755 (PMC12346285; doi:10.3390/foods14152755)
Supplement: Supplementary file 1 [file foods-14-02755-s001.zip › foods-3764597-supplementary.pdf]

A mixed-method assessment of drivers and barriers for substituting dairy with plant-based alternatives by Danish adults

Beatriz Philippi Rosane [1,2,□,*] , Lise Tjørring [3,□], Annika Ley [4], Derek V. Byrne [2,5], Barbara Vad Andersen [2,5], Susanne Gjedsted Bügel [1,2], and Sophie Wennerscheid [6]

[1] Department of Nutrition, Exercise and Sports, University of Copenhagen, Rolighedsvej 26, 1980 Frederiksberg, Denmark; bpr@nexs.ku.dk; shb@nexs.ku.dk.

2 Sino-Danish College (SDC), University of Chinese Academy of Sciences, 380 Huaibeizhuang, Huairou District, Beijing, China

3 Queen Mary's Center, University of Copenhagen, Øster Farimagsgade 3, Building 30, 1353 Copenhagen K, Denmark; alt@samf.ku.dk.

4 Department of Food · Nutrition · Facilities, FH Münster, Corrensstraße 25, 48149 Münster, Germany annika.ley@freenet.de.

5 Food Quality Perception and Society Team, iSense Lab, Department of Food Science, Faculty of Technical Sciences, Aarhus University, Agrofood park 48, 8200 Aarhus, Denmark; derekv.byrne@food.au.dk; barbarav.andersen@food.au.dk

6 Department of Nordic Studies and Linguistics, University of Copenhagen, Emil Holm's Channel 2, 2300 Copenhagen S, Denmark; sophie.wennerscheid@hum.ku.dk

[□] Shared first authorship.

[*] Correspondence: bpr@nexs.ku.dk.

Supplementary Materials

SM 1. List of plant-based products used in the intervention.

Table S1. Plant-based products used in the intervention and their nutritional information (given for 100mL for drinks and 100g for yoghurt and cheese alternatives).

Product	Ingredients	Energy (kJ)	Fats (g)	SFAs (g)	MUFAs (g)	PUFAs (g)	Carbs (g)	Sugars (g)	Fibres (g)	Protein (g)	Salt (g)	Calcium (mg)	Vitamin D (µg)	Vitamin B2 (µg)	Vitamin B12 (µg)
**Alternatives to milk (brand: Dryk)**															
Oat drink	oat base (water, oats 10%), rapeseed oil, calcium phosphate, salt (iodized salt), vitamins (D3, riboflavin and B12)	206	1.2	0.1	NA	NA	8.5	3.7	NA	0.9	0.1	120	1	0.21	0.38
Pea Drink	water, pea protein (2.5%), rapeseed oil, sugar, acidity regulator (dipotassium	176	2.3	0.2	NA	NA	1.5	1.5	NA	2	0.2	120	1	0.21	0.38

	phosphate), (calcium carbonate, calcium phosphate), gluten-free oat oil, salt, vitamins (D3, riboflavin and B12)														
Chocolate oat drink	Oat base (water, oats 10%), sugar, cocoa powder 1%, rapeseed oil, calcium (calcium carbonate, calcium phosphate), salt (added iodine), flavourings, vitamins (D3, riboflavin and B12)	269	1.3	0.2	NA	NA	11	7.1	NA	1.2	0.2	120	1	0.21	0.38
**Alternatives to yoghurt (brand: Alpro)**															
Plantgurt natural	water, shelled soybeans (7.9%), sugar, tricalcium citrate, stabilizer (pectin), acidity regulators (sodium citrate, citric acid), sea salt, antioxidants (tocopherol-rich extract, ascorbic acid esters of edible fatty acids), vitamins (B12, D2), yogurt cultures (S . thermophilus, L. bulgaricus).	210	2.3	0.4	0.5	1.4	2.1	2.1	1	4	0.3	120	0.75	NA	0.38
Plantgurt blueberry	water, sugar, shelled soybeans (7.2%), (blueberries (5.9%)), glucose-fructose syrup, tricalcium citrate, acidity regulators (sodium citrate, citric acid), stabiliser (pectin), natural flavouring, hibiscus and carrot juice concentrate, vitamins (riboflavin (B2), B12, D2), sea salt, antioxidants (tocopherol-rich extract, ascorbic acid esters of edible fatty acids), yoghurt cultures (S. thermophilus, L. bulgaricus)	322	2	0.3	0.4	1.2	10.1	10.1	0.9	3.6	0.2	120	0.75	NA	0.38
Mango Greek style plantgurt	water, mango (17.8%) (juice from concentrate (11.1%), fruit (6.7%)), shelled SOYBEANS (9.7%), tricalcium citrate, stabilizer (pectins), acidity regulators (sodium citrates, citric acid), natural flavoring, modified starch, sea salt, antioxidants (tocopherol-rich extract, ascorbic acid esters of fatty acids), carrot extract, pumpkin extract, vitamins (riboflavin (B2), B12, D2), yogurt cultures (S. thermophilus, L. bulgaricus)	257	2	0.4	0.4	1.2	5.8	5.8	1	3.7	0.22	120	0.75	NA	0.38
**Alternatives to cheese (brand: Casheury)**															
Bianco[1]	Cashew nuts*, water, coconut oil*, lemon*, onion powder*, sea salt, lactic acid	1841	39	21	NA	NA	14	3	NA	9.2	1	NA	NA	NA	NA

Cashewmozza[1]	Cashew nuts*, coconut oil*, water, nutritional yeast, psyllium husk, sea salt, lemon*, souring culture	1841	39	21	NA	NA	14	3	NA	9.2	1	NA	NA	NA	NA
Ramslök[2]	Cashew nuts* 40%, water, coconut oil, onion powder*, sea salt, dried wild garlic*, garlic*, parsley*, black pepper*, souring culture	2468	46.5	9.2	NA	NA	31.5	2	0	15.3	1.5	NA	NA	NA	NA
Bruschetta[2]	Cashew nuts* 40%, coconut oil*, water, sea salt, *lemon, *tomato, *oregano, *garlic, *onion, *scallion, *smoked paprika, *basil, souring culture	2468	46.5	9.2	NA	NA	31.5	2	0	15.3	1.5	NA	NA	NA	NA

[1]Cashew alternatives to fermented cheese; Bianco is a white cheese for everyday use on toast or sandwiches; Casheumozza is an alternative to fresh mozzarella and can be used in pizzas or grilled sandwiches.

[2]Cashew alternatives to aged cheese; Ramslök is a white creamy cheese flavoured with Swedish wild garlic; Bruschetta is a white creamy cheese flavoured with tomatoes and Mediterranean herbs.

*Organic ingredients.

NA: information not available

SM 2. Social questions added to the questionnaire.

Table S2. Questionnaire additional questions

**English**	**Danish**
1. How big a role does your social circle (friends, family, partner, etc.) have for your choice of food?	1. Hvor stor en rolle spiller din sociale omgangskreds (familie, venner, kæreste osv.) for dit madvalg?
2. How big a role does social media (online recipes, Instagram pictures of food, Facebook posts, etc) have in your choice of food?	2. Hvor stor en rolle spiller sociale medier (online opskrifter, instagram-billeder af mad, facebook-opslag osv.) for dit madvalg?
3. How big a role do film, TV series, literature, art, and music play in your perception of the climate crisis?	3. Hvor stor en rolle spiller film, tv-serier, litteratur, kunst og music for din opfattelse af klimakrisen?
Likert scale:<br>Nothing/not at all<br>A little<br>Some<br>Much<br>Very much	Likert scale:<br>Ikke noget<br>Lidt<br>Noget<br>Meget<br>Rigtig meget

SM 3. Description of the reflection and writing workshop on dairy attachment and plant-based alternatives

Two weeks after the dietary intervention started, we invited ten of the twenty participants to a three-hour workshop, where they were provided with textual and visual materials on milk production and consumption and encouraged to reflect on various milk-related topics and issues. The workshop had two main goals. First, we wanted to offer participants the opportunity to reflect on their eating habits, as well as the underlying values, memories and traditions. The second goal was tied to our research setup. Based on the assumption that collective reflection and social interaction can positively influence transformation processes, the workshop was designed to gather empirical data to test our hypothesis. Would the participants who attended the workshop be more motivated to shift their diet?

The workshop was organized by Lise Tjørring and Sophie Wennerscheid.

At the beginning of the workshop, we introduced ourselves, explained our academic background and specific role in the study, and set out the structure and aim of the reflection workshop. We emphasized that there is room for everyone; all opinions, ideas, concerns, feelings are allowed. We don't judge but want an open dialogue. We explain that we will use the data from the workshop in our study. Thereafter, the participants introduce themselves (Why are they participating? What is their motivation? Expectations, possible concerns, questions?)

The material used and the specific reflection and writing tasks we asked the participants to complete were as follows:

Writing exercise 1: Dairy attachment

Inspirational material: an excerpt from Tove Ditlevsen's novel The Street of Childhood [57]) on two young girls reflecting on the difference between butter and margarine.

Writing task: What comes to your mind when thinking of dairy products? Write a brief text on a childhood memory involving dairy products, focusing on the concrete setting, taste, and emotions associated with consuming dairy products.

Reflection exercise: milk in Danish food culture

To encourage the participants to reflect on the role of milk in Danish food culture, we gave them an article by food historian Bettina Buhl, asked them to read and discuss the text in small groups, and then presented their thoughts in the plenum.

Task: Read and discuss the text "Milk is not sustainable, but it is part of our culture" [33] in groups of two to four and present the main points of the text.

Source: https://vidogsans.dk/maelk-er-ikke-baeredygtig-men-den-er-en-del-af-vores-kultur/

Writing exercise 2: Milk production and animal welfare

To give a better understanding of milk production, animal welfare, and humans relation to dairy cows we watched the short film Fabrikken Caroline (1951) [58] and read a text by anthropologist Sofie Isager Ahl. Based on this, we asked the participants to write a short text from the perspective of a dairy cow today. The purpose of this exercise was to challenge the typical, anthropocentric viewpoint and to foster empathy for a dairy cow.

Sources: https://www.dfi.dk/viden-om-film/filmdatabasen/film/fabrikken-caroline

Sofi Isager Ahl: “Komødre” (Cow mothers”, from her book Regenration (2023). [59]

Writing task: Write a text from a cow’s perspective. Put yourself in the animal’s place and reflect on what a cow might experience.

Writing exercise 3: Challenges and opportunities of plant-based dairy alternatives

We asked the participants to reflect on both possible challenges and opportunities when shifting from dairy to plant-based alternatives by writing down their thoughts.

Writing task: Write a short text about possible conflicts and potentials when shifting to plant-based alternatives. What happens when I switch from milk, etc., to plant-based alternatives? How do family, friends, colleagues react? Which conflicts or uncomfortable situations could arise? How will you handle it? What positive things could happen?

Writing Exercise 4: Sustainability

Ultimately, we asked the participants about their general attitudes and thoughts about the sustainability of dairy and plant-based alternatives.

Writing task: Write a brief text about your thoughts on sustainability regarding plant-based alternatives.

References:

57. Ditlevsen T (1943) Barndommens Gade [The Street of Childhood], 1st ed. Copenhagen

58. Melson S (1951) Fabrikken Caroline [The Factory Caroline]. Statens Filmcentral, Denmark.

59. Ahl, Sofie Isager. 2023. Komødre. In Regeneration: gensidigt helendepraksisser i en ny jordbrugsbevægelse (pp. 51–86). Laboratorietfor Æstetik og Økologi

SM 4. Semi-structured qualitative interview guide, translated from Danish.

Describe your experience of participating in the pilot project.

What motivated you to join the project?

Describe a typical day with a focus on food and meals.

- What do you eat, and when?
- What is a good meal for you?
- Do you eat alone/with others?
- Why do you choose to eat what you do?

What was your experience of taking part in the social track of the pilot project?

- What did you get out of being together with others in the project?
- Did it lead to new reflections? Which ones? What have these reflections meant to you?

How did your social circle react to your transition to consuming plant-based products?

- Reactions from family, friends, and colleagues?
- Did you experience new possibilities or conflicts?
- How did you feel about their reactions?
- What has it meant to you that this was a pilot project and not necessarily a permanent transition?
- What do you think people generally think about those who eat plant-based food?

Do you know others who have transitioned to plant-based food?

- What have their experiences been socially and health-wise?
- Why did they make the change?

What are your childhood memories of food?

- What food habits have you brought with you from your childhood? What have you changed in terms of eating since your childhood? What food habits would you like to pass on to future children?
- Do you have any specific memories about dairy products?
- Your parents' attitudes towards food/meals?

What is your perception of dairy and the plant-based alternatives?

- Taste?
- Price?
- Information on the packaging?
- Bodily effects/experience?
- Other things?

What is “a sustainable food choice” to you?

- What does it specifically entail in your daily life?
- What is difficult/easy about it?
- How important is sustainability in your everyday life?

What are your thoughts about your responsibility for the green transition?

- Individual responsibility vs. political responsibility?
  - What do you do/don’t do?
- What are your perceived opportunities and limitations in the green transition?

Have you noticed any changes or developments over the four weeks regarding:

- Taste?
- Habits?
- Bodily effects/experiences?
- Opinions and perspectives (e.g., regarding sustainability)?

What are your future plans regarding food?

- Are you going to continue eating plant-based? Why/why not?
- What direction would you like to see our “food system” take?

May I have a tour of your kitchen and dining area?

SM 5. Food group classifications and standardized portion sizes.

One aim of the dietary intervention was to investigate potential nutritional changes in participants' diets due to substituting dairy products. This was achieved by comparing dietary data collected before and after the intervention. Data preparation involved reviewing dietary registrations and transferring raw data from the Research Electronic Data Capture (RedCap) system into the nutritional software Vitakost©, Vitakost Aps 2024. The processed data was then exported into Microsoft Excel 365 for analysis. Dietary intake was categorized into food groups, following definitions from the Danish Reference for portion sizes [60] or with standardized portion sizes provided by Vitakost©. For more detailed analysis, some food groups, like dairy, were further subdivided (i.e. milk, yoghurt, cheese, and other dairy products) to observe the relative contribution of each category to overall dairy consumption. If no subcategory was defined, the main food group corresponded directly to the subcategory. All food groups, along with their respective subcategories, are presented in Table S3.

Table S3. Classification of the food groups into their sub-categories including the minimum and maximum portion sizes.

		Range of Portion Sizes			
		Minimum		Maximum	
**Food Group**	**Subgroup**	**Food**	**g**	**Food**	**g**
**Fruit and Fruit Products**	Fruit and Fruit Products	Raspberry	4	Honey Melon	370
**Vegetables and Vegetable Products**	Vegetables and Vegetable Products	Chives	2	Beetroot Soup, Ready to eat	400
**Herbs and Spices**	Herbs and Spices	Ginger powder (pinch)	0,1	Mixed Herbs	20
**Legumes**	Legumes	Snap Peas	25	Yellow Daal, Ready to Eat	325
**Potatoes**	Potatoes	Potato, Raw	60	Potato, baked	150
**Potatoes**	Potato Products	French Fries	100	Gnocchi	250
**Nut and Seeds**	Nut and Seeds	Psyllium Husks	1,5	Peanuts; Walnuts; Mixed Nuts	40
**Whole Grains**	Whole Grains	Wheat Bran	4,5	Bulgur, Cooked	240
**Whole Grains**	Bread from Whole Grains	Rye Bread with sunflower seeds	40	Bun (Oat/ Rye/ Whole Wheat)	62,5
**Whole Grains**	Products from Whole Grains	Cereal Bar	50	Whole Wheat Spaghetti	150
**Cereals**	Cereals	Rice, Raw	60	Rice, cooked	125
**Cereals**	Bread from Refined Cereal	Crisp Bread	13	Rosemary Foccacia	300
**Cereals**	Products from Refined Cereal	Rice Cake	7	Tortellini	250

**Cereals**	Sweet Pastries	Cookie	8	Rice pudding	250
**Pseudocereal**	Pseudocereal	Quinoa, Raw	50	Quinoa Salad, Ready to eat	275
**Dairy**	Milk	Milk (in Scrambled Eggs)	10	Protein Drink	500
**Dairy**	Yoghurt	Yoghurt (all types)	200	Yoghurt (all types)	200
**Dairy**	Cheese and Cheese Products	Parmesan	10	Feta; Halloumi	30
**Dairy**	Other Dairy Products	Butter (for cooking/ frying)	5	Shake (Fast Food)	500
**Plant-based Dairy**	Plant-Based Milk	Plant Based Milk (in coffee)	35	Coconut Milk	382
**Plant-based Dairy**	Plant Based Yoghurt	Plant-based yoghurt (all types)	200	Plant-based yoghurt (all types)	200
**Plant-based Dairy**	Plant-Based Cheese	Plant-based cheese (all types)	20	Plant-based cheese (all types)	20
**Plant-based Dairy**	Other Plant-Based Dairy Products	Vegan Butter	14	Vegan Cream	50
**Meat**	Red Meat	Minced Meat	100	Braised Beef Cheek	150
**Meat**	White Meat	Chicken Skewer	80	Chicken in Curry	340
**Meat**	Processed Meat	Pepperoni	5	Chicken Meatball; Minced Meat Patty	100
**Meat Substitutes**	Meat Substitutes	Vegan Spread/ Cuts	10	Carrot Beef; Vegan Minced Beef	100
**Fish and Shellfish**	Fish and Shellfish	Smoked Salmon Slices	20	Steamed fish/ cold-smoked salmon	569
**Eggs**	Eggs	Egg Yolk	25	Fried Egg	65
**Fats and Oils**	Fats and Oils	Oil (all types)	10	Oil (all types)	10
**Sweets**	Sweets	Chocolate Spread; Chocolate (all types)	4	Chocolate Mousse (Powder)	92
**Salty Snacks**	Salty Snacks	Salted Popcorn	20	Chips (all types)	25
**Sauces**	Cold Sauces	Mayonnaise (all types), Remoulade	7	Ketchup	20
**Sauces**	Hot Sauces	Pesto	18,3	Vegan Bolognese	180
**Non-Alcoholic Beverages**	Water	Water (all types)	200	Water (all types)	200

**Non-Alcoholic Beverages**	Fruit Juice	Fruit Juice (all types)	200	Fruit Juice (all types)	200
**Non-Alcoholic Beverages**	Sugary Beverages	Coke; Lemonade; Pepsi; Soda	200	Energy Drink	250
**Non-Alcoholic Beverages**	Low Caloric Beverages	Espresso	30	Energy Drink, Sugar Free	500
**Non-Alcoholic Beverages**	Other Non-Alcoholic Beverages	Matcha Powder	2	Alcohol-Free Beer	355
**Alcoholic Beverages**	Beer	Beer, Tuborg	200	Beer, IPA	750
**Alcoholic Beverages**	Wine	White Wine	146	White Wine	146
**Alcoholic Beverages**	Other Alcoholic Beverages	Cocktail	211	Gin	9,4

The data analysis phase utilized these food groupings to calculate portion sizes, enabling comparison not just in grams or kilocalories, but by portions—important given the differing densities of foods, particularly between dairy and plant-based alternatives. Portion sizes were determined by dividing the amount of food or drink consumed by its standardized portion size. Nutritional analysis was conducted using Vitakost, which provided averages for energy intake, macronutrients, micronutrients, and vitamins per participant and overall. Additionally, the top three food sources per nutrient were identified, allowing the study to assess which foods primarily contributed to each nutrient's intake and to evaluate the role of dairy or plant-based dairy substitutes. The average intake of food groups and subcategories was calculated and converted to relevant units (grams, kJ, MJ, kJ per 10 MJ, and kcal per 10 MJ). Results were compared with the Nordic Nutrition Recommendations [61] and dietary habits of the Danish population from the report Dietary Habits in Denmark 2011-2013 [26]

References 26. Pedersen, A.N.; Christensen, T.; Matthiessen, J.; Knudsen, V.K.; Rosenlund-Sørensen, M.; Biltoft-Jensen, A.; Hinsch, H.-J.; Ygil, K.H.; Kørup, K.; Saxholt, E.; et al. *Dietary Habits in Denmark 2011–2013*; National Food Institute: Kongens Lyngby, Denmark, 2015; ISBN 978-87-93109-39-1.

60. Ygil, K. H. (2013). Mål, vægt og portionsstørrelser på fødevarer. (1 ed.) DTU Fødevareinstituttet. Online available at www.food.dtu.dk

61. Nordic Council of Ministers. (Ed.) (2023). Nordic Council Nordic Nutrition Recommendations. Online available at https://pub.norden.org/nord2023-003.

SM 6. Results of motivation to eat climate-friendly

Table S4. Results of questionnaire items and answers related to motivation to eat climate-friendly regarding product characteristics.

Questionnaire item	Baseline (mean ± SD)	End of Intervention (mean ± SD)
How important are the following elements for your motivation to eat climate-friendly?		

That the product is low price	66.18 ± 29.87	68.88 ± 25.16
That the product is healthy and nutritious	79.5 ± 17.15	78.25 ± 12.69
That the product has sufficient product information on the packaging (e.g., ingredients list, labels, production method)	69.5 ± 21.75	63.75 ± 17.93
That the product has a low impact on climate	66.5 ± 17.99	69.94 ± 15.47
The product's sensory properties (appearance, smell, taste and/or texture)	77.44 ± 21.62	75.50 ± 11.21
The product's functionality in common/everyday dishes	74.31 ± 14.46	70.50 ± 11.21
That the product is easy to prepare/use	67.69 ± 17.76	69.56 ± 13.48
That there are several variants of the products to choose from	44.69 ± 21.65	50.25 ± 24.50
That the product can function in my habitual diet	65.13 ± 19.72	70.69 ± 18.60
That the product is familiar to me	36.63 ± 18.60	41.94 ± 19.59
That the product is new to me	22.69 ± 18.96	29.13 ± 21.17
Do you think that climate-friendly food products are challenged in any of these aspects? (check all that apply)	N of participants who checked<br>Price - 10<br>Health and nutrition - 3<br>Product information - 4<br>Climate impact - 1<br>Sensory properties - 9<br>Functionality in everyday dishes - 6<br>Preparation requirements/user friendliness - 4<br>Variants to choose from - 2<br>Familiarity - 3<br>Novelty - 4	N of participants who checked<br>Price - 13<br>Health and nutrition - 5<br>Product information - 4<br>Climate impact - 0<br>Sensory properties - 8<br>Functionality in everyday dishes -5<br>Preparation requirements/user friendliness - 3<br>Variants to choose from - 2<br>Familiarity - 2<br>Novelty – 2

SM 7. Top 3 food sources of nutrients.

On **Table S5** we have compiled the top 3 food sources of different nutrients to investigate if the changes in diet composition were solely driven by the dietary intervention or if the intake of other foods explained some of the changes.

Table S5. Top 3 foods source of nutrient, per nutrient per assessment (Baseline and End of Intervention).

TOTAL average (Vitakost results)	Baseline	Top 3 Foods/ Source		End Intervention	Top 3 Foods/ Source	
		**Food**	**Total per Food and Unit**		**Food**	**Total per Food and Unit**
kcal	2488.0	Pizza Dough	5048.0	2139.0	Oat Drink	3498.0
		Beer	4189.0		Rye Bread	3363.0
		Skimmed Milk	2818.0		Oatmeal	2421.0
Protein (g)	92.5	Skimmed Milk	214.0	75.3	Plant based Yoghurt, Natural	173.0
		Chicken	162.0		Chicken Breast	158.0
		Mozzarella	160.0		Rye Bread	94.9
Carbohydrate (g)	287.0	Pizza Dough	914.0	248.0	Rye Bread	633.0
		Rye Bread	483.0		Oat Drink	595.0
		Müsli	328.0		Oatmeal	382.0
Sugars (g)	108.0	Beer	311.0	74.4	Banana	266.0
		Skimmed Milk	287.0		Oat Drink	259.0
		Chocolate Milk	239.0		Apple	121.0
Dietary fibers (g)	29.7	Rye Bread	114.0	38.2	Rye Bread	150.0
		Chia Seeds	65.0		Rye Bread Crunch	87.0
		Rye Bread (Sunflower Seeds)	50.8		Chia Seeds	74.0
Fat (g)	90.2	Butter	274.0	80.8	Olive Oil	240.0
		Olive Oil	198.0		Plant-based Cheese, Wild Garlic	177.0
		Mozzarella	139.0		Plant-based Cheese, Bianco	144.0
Saturated fatty acids (g)	37.9	Butter	174.0	20.4	Coconut Milk	79.2
		Coconut Milk	95.7		Plant-based Cheese, Bianco	77.7
		Mozzarella	90.3		Plant based Cheese, Mozzarella	41.4
Cholesterol (mg)	198.0	Eggs	2007.0	132.0	Eggs	2529.0
		Fried Eggs	673.0		Chicken Breast	511.0
		Chicken	626.0		Mayonnaise	286.0
Omega 3 (g)	1.6	Chia Seeds	30.6	1.9	Smoked Salmon	10.8
		Mayonnaise	4.8		Salmon Filet	9.1
		Walnuts	3.4		Mayonnaise	7.8
Calcium, Ca (mg)	1172.0	Skimmed Milk	7454.0	792.0	Plant-based Yoghurt, Natural	5185.0
		Water	6200.0		Water	4856.0
		Mozzarella	5004.0		Pea Drink	2998.0
Iron, Fe (mg)	9.2	Mixed Herbs	32.4	9.7	Rye Bread	27.0
		Rye Bread	20.6		Oat Meal	25.3
		Oatmeal	16.8		Mixed Herbs	19.3
Iodine, I (µg)	846.0	Water	641.0	240.0	Algae	7200.0
		Skimmed Milk	623.0		Fish Meatballs	599.0
		Beer	473.0		Water	502.0

Magnesium, Mg (mg)	337.0	Water	1033.0	312.0	Oatmeal	1017.0
		Beer	865.0		Rye Bread	816.0
		Coffee	773.0		Water	809.0
Phosphorous, P (mg)	1330.0	Skimmed Milk	5866.0	842.0	Oatmeal	2886.0
		Cheese Slices	2746.0		Rye Bread	2714.0
		Mozzarella	2711.0		Chia Seeds	1849.0
Salt, NaCl	5.8	Rye Bread	15.2	5.5	Rye Bread	20.0
		Pizza Dough	14.3		Plant-based Yoghurt, Natural	10.8
		Minced Beef	13.8		Foccacia, Rosmarin	7.8
Selenium (µg)	30.8	Greek Yoghurt	130.0	37.1	Chicken Breast	160.0
		Eggs	126.0		Eggs	159.0
		Skimmed Milk	94.1		Pearls Barley	136.0
Zinc, Zn (mg)	8.7	Skimmed Milk	25.2	6.2	Rye Bread	23.6
		Mozzarella	22.2		Oatmeal	19.6
		Minced Beef	21.4		Pasta	10.0
Vitamin B2 (Riboflavin) (mg)	1.5	Skimmed Milk	10.4	1.2	Collagen Powder	7.0
		Beer	3.5		Pea Drink	5.3
		Milk Shake	3.3		Eggs	3.0
Vitamin B12 (µg)	3.1	Skimmed Milk	29.9	3.6	Liver Pate	20.2
		Minced Beef	10.8		Energy Drink	20.0
		Mozzarella	9.6		Smoked Salmon	16.5
Vitamin B12 (added) (µg)	0.0	/		0.8	Plant-based Yoghurt, Natural	16.4
		/			Energy Drink, Red Bull	9.8
		/			Plant Based Yoghurt, Blueberry	4.0
Vitamin C (mg)	95.6	Red Pepper	542.0	91.8	Green Kale	676.0
		Broccoli	449.0		Red Pepper	287.0
		Cabbage, pointed	310.0		Broccoli	242.0
Vitamin D3 (µg)	0.7	Chicken	10.8	1.7	Salmon Filet	23.6
		Eggs	7.0		Smoked Salmon	16.5
		Smoked Salmon	3.0		Herring, Curry	8.8

SM 8. Total energy intake and energy consumption per food group

Table S6 Total average daily energy intake and per food group are normalized by 10MJ daily intake. %E represents the contribution of that food group to total energy intake.

	**Baseline**		**End** of **Int**ervention	
**Food Group (kJ/ 10 MJ)**	**Energy (kJ/ 10 MJ)**	**%E**	**Energy (kJ/ 10 MJ)**	**% E**
Total daily energy intake	1038.01 ± 2780.33	-	8953.70 ± 2493.42	-
Fruits and Fruit Products	557.58 ± 487.27	4.55	513.20 ± 489.33	5.62
Vegetables	337.57 ± 225.27	2.76	308.47 ± 177.83	3.38
Herbs and Spices	27.70 ± 46.65	0.23	19.53 ± 29.91	0.21
Legumes	369.08 ± 440.15	3.01	225.72 ± 296.68	2.47
Potatoes and Potato Products	245.80 ± 323.21	2.01	121.43 ± 247.13	1.33
Nuts and Seeds	258.23 ± 281.44	2.11	552.48 ± 718.71	6.05
Whole-grain cereals	1242.22 ± 850.28	10.14	1490.07 ± 806.29	16.32
Cereals and Pseudocereals	2003.30 ± 924.71	16.35	1518.77 ± 838.73	16.63
Dairy (total)	2236.71 ± 1199.90	18.26	204.55 ± 385.62	2.24
Milk	672.66 ± 986.32	5.49	0.00 ± 0.00	0.00
Yogurt	247.01 ± 986.32	2.02	26.90 ± 78.45	0.29
Cheese	795.07 ± 625.41	6.49	57.13 ± 203.80	0.63
Other Dairy Products	521.96 ± 396.24	4.26	120.52 ± 297.91	1.32
Plant-based Dairy Alternatives (total)	136.89 ± 257.46	1.12	1834.20 ± 1140.69	20.09
Plant-based Milk Alternatives	35.66 ± 61.62	3.44	585.54 ± 461.38	5.15
Plant-based Yogurt Alternatives	25.37 ± 53.59	2.44	331.48 ± 424.34	4.74
Plant-based Cheese Alternatives	25.37 ± 0.00	2.44	217.80 ± 149.36	1.67
Other plant-based products	0.00 ±0.00	0.00	32.44 ± 31.39	0.35
Meats (total)	474.90 ± 683.97	3.88	307.38 ± 412.50	3.37
Red Meat	52.93 ± 205.00	0.43	32.75 ± 115.58	0.36
White Meat	202.63 ± 523.21	1.65	161.58 ± 359.62	1.77
Processed Meat	219.35 ± 294.37	1.79	113.05 ± 294.61	1.24
Meat Substitutes	67.85 ± 128.43	0.55	28.47 ± 68.32	0.31
Fish and Shellfish	77.62 ± 131.59	0.63	251.40 ± 344.70	2.75
Eggs	101.50 ± 119.08	0.83	104.69 ± 154.60	1.15
Fats and Oils	155.67 ± 170.39	1.27	287.17 ± 360.77	3.15
Sweets	698.31 ± 524.55	5.70	419.37 ± 511.69	4.59
Salty Snacks	144.50 ± 299.16	1.18	46.76 ± 192.67	0.51
Sauces	215.07 ± 237.56	1.76	195.34 ± 239.50	2.14
Non-Alcoholic Beverages	135.45 ± 184.60	1.11	174.49 ± 171.97	1.91
Alcoholic Beverages	51.41 ± 73.49	0.42	14.65 ± 37.78	0.16

SM 9. Participants self-reported overall liking for PBDA and frequency of consumption.

Table S7. Results of questionnaire items and answers regarding overall like of PBDA and consumption.

What/when	Baseline	End of intervention
Tried plant-based milk	15 tried	16 yes
Frequency intake of plant-based milk	Never 5<br>Once a week 7<br>2-6 times a week 2<br>Once a day 1<br>2-3 times a day<br>4-5 times a day	Never 1<br>Once a week 0<br>2-6 times a week 5<br>Once a day 1<br>2-3 times a day 4<br>4-5 times a day 4
Favourite plant-based milk	Soy 0<br>Coconut 2<br>Rice 0<br>Cashew nut 0<br>Almond 2<br>Pea 2<br>Oat 8<br>Other 1	Soy 0<br>Coconut 0<br>Rice 0<br>Cashew nut 0<br>Almond 2<br>Pea 7<br>Oat 7<br>Other 0
Liking plant-based milk (mean ± SD)	64 ±16.84	65.87 ± 17.17
Tried plant-based cheese	8 yes	16 yes
Frequency intake of plant-based cheese	Never 7<br>Once a week 1<br>2-6 times a week<br>Once a day<br>2-3 times a day<br>4-5 times a day	Never 2<br>Once a week 4<br>2-6 times a week 6<br>Once a day 0<br>2-3 times a day 2<br>4-5 times a day 2
Liking plant-based cheese (mean ± SD)	31.87 ± 21.37	47.93 ± 25.93
Tried plant-based yoghurt	8 yes	16 yes
Frequency intake of plant-based yoghurt	Never 7<br>Once a week 1<br>2-6 times a week<br>Once a day<br>2-3 times a day<br>4-5 times a day	Never 0<br>Once a week 2<br>2-6 times a week 9<br>Once a day 2<br>2-3 times a day 1<br>4-5 times a day 2
Favourite plant-based yoghurt	Soy 4<br>Coconut 0<br>Rice 0<br>Cashew nut 0<br>Almond 1<br>Pea 0<br>Oat 1<br>Other 2	Soy 10<br>Coconut 0<br>Rice 0<br>Cashew nut 0<br>Almond 0<br>Pea 1<br>Oat 3<br>Other 0
Liking plant-based yoghurt (mean ± SD)	63.25 ± 25.62	73.5 ± 22.42
